# Encapsulation and Controlled Release of Resveratrol Within Functionalized Mesoporous Silica Nanoparticles for Prostate Cancer Therapy

**DOI:** 10.3389/fbioe.2019.00225

**Published:** 2019-09-18

**Authors:** Zanib Chaudhary, Sugarniya Subramaniam, Gul Majid Khan, Muhammad Mustafa Abeer, Zhi Qu, Taskeen Janjua, Tushar Kumeria, Jyotsna Batra, Amirali Popat

**Affiliations:** ^1^School of Pharmacy, The University of Queensland, Brisbane, QLD, Australia; ^2^Department of Pharmacy, Quaid-i-Azam University, Islamabad, Pakistan; ^3^School of Biomedical Sciences, Queensland University of Technology, Brisbane, QLD, Australia; ^4^Faculty of Health, Institute of Health and Biomedical Innovation, Australian Prostate Cancer Research Centre-Queensland, Queensland University of Technology, Brisbane, QLD, Australia; ^5^Translational Research Institute, Woolloongabba, QLD, Australia; ^6^Mater Research Institute, Woolloongabba, QLD, Australia

**Keywords:** resveratrol, mesoporous silica nanoparticles, chemotherapy, anti-cancer activity, prostate cancer

## Abstract

Resveratrol (RES) is a naturally existing polyphenol which exhibits anti-oxidant, anti-inflammatory, and anti-cancer properties. In recent years, RES has attracted attention for its synergistic effect with other anti-cancer drugs for the treatment of drug resistant cancers. However, RES faces the issues of poor pharmacokinetics, stability and low solubility which limits its clinical application. In present study, RES has been loaded onto uniformly sized (~60 nm) mesoporous silica nanoparticles (MSNs) to improve its *in vitro* anti-proliferative activity and sensitization of Docatexal in hypoxia induced drug resistance in prostate cancer. RES was efficiently encapsulated within phosphonate (negatively charged) and amine (positively charged) modified MSNs. The effect of surface functionalization was studied on the loading, *in vitro* release, anti-proliferative and cytotoxic potential of RES using prostate cancer cell line. At pH 7.4 both free and NH_2_-MSNs loaded RES showed burst release which was plateaued with almost 90% of drug released in first 12 h. On the other hand, PO_3_-MSNs showed significantly slower release kinetics with only 50% drug release in first 12 h at pH 7.4. At pH 5.5, however, both the PO_3_-MSNs and NH_2_-MSNs showed significant control over release (around 40% less release compared with free RES in 24 h). Phosphonate modified MSNs significantly enhanced the anti-proliferative potential of RES with an IC_50_ of 7.15 μM as compared to 14.86 μM of free RES whereas amine modified MSNs didn't affect proliferation with an IC_50_ value higher than free RES (20.45 μM). Furthermore, RES loaded onto PO_3_-MSNs showed robust and dose dependent sensitization of Docatexal in hypoxic cell environment which was comparable to pure RES solution. This study provides an example of applicability of MSNs loaded with polyphenols such as RES as next generation anticancer formulations for treating drug resistant cancers such as prostate cancer.

## Introduction

Prostate cancer is the leading cause of cancer-related deaths in men over the age of 50 (Siegel et al., 2011). Moreover, due to the prolonged latency of prostate cancer, it has become an attractive target for chemotherapeutic strategies (Van Poppel and Tombal, 2011). Hypoxia is a common event in solid tumor environment which leads to distant metastasis, resistance to chemotherapeutics and epithelial-mesenchymal transition (EMT) (Semenza, 2012; Liu et al., 2014; Rankin and Giaccia, 2016). The underlying mechanism involves the role of hypoxia inducible factor (HIF) which regulates the expression of proteins that contribute to the oncogenesis and enable the cancer cells to survive under hypoxic conditions (Vaupel, 2004). Docetaxel (Doc) has been considered as a standard first line therapy in prostate cancers (Francini and Sweeney, 2016; Mansour et al., 2018; Singh et al., 2018). However, the survival rate remains only modest owing to rapid resistance to Doc acquired by patients (Qian et al., 2018). There is a dire need to revamp the mainstay treatment of prostate cancer i.e., Doc to target hypoxia-induced chemoresistance (Seruga et al., 2010; Fernandez et al., 2015).

Recently, many researchers have explored the antimetastatic efficacy of dietary polyphenols *in vivo* and their use as a supplementary therapy is gaining momentum (Hussain et al., 2016; Amawi et al., 2017). Resveratrol (RES), a naturally occurring polyphenol, exhibits a wide range of properties such as anti-inflammatory, anti-oxidant, pro-apoptotic, and anti-angiogenic effects (Kundu and Surh, 2008; Lancon et al., 2016; Eräsalo et al., 2018). Studies suggest that the dietary intake of RES could prevent cancers and, enhance the efficacy of chemotherapeutic agents (Khan et al., 2008). In experimental models, RES has shown anti-cancer effects and has shown to significantly suppress the initiation and progression of tumor growth (Khan et al., 2008; Yu et al., 2012). RES has been previously reported to have anti-cancer effects on prostate cancer cell lines LNCaP, DU145, and PC3 (Athar et al., 2009; Cimino et al., 2012). Although, RES offers many benefits its effectiveness is decreased by its low bioavailability and serum stability (De Vries et al., 2018; Huang et al., 2019; Tabibiazar et al., 2019). Many drug delivery systems including nanoparticles have been used in order to improve its stability, bioavailability and targetability (Summerlin et al., 2016; Huang et al., 2019).

In theory, the nanoparticles should load high contents, protect the cargo until it reaches the site of action, allow efficient uptake of the loaded/encapsulated drug by the cells and escape reticuloendothelial system (Watermann and Brieger, 2017). These features are required to overcome challenges such as multidrug resistance to chemotherapeutic drugs worsening morbidity of the cancers. The current development of mesoporous silica nanoparticles (MSNs) strongly advocates that MSNs can overcome these barriers owing to their unique features (He and Shi, 2014). These features include flexibility in surface functionality due to silanol functional groups available for facile modification, superior textural properties such as pore size, surface area and pore volume of mesopores and controllable morphology (Zhou et al., 2018). Therefore, silica nanoparticles have been recently used in a human trial for patients with melanoma and have shown strong implications in cancer diagnosis (Phillips et al., 2014). An interesting feature of MSNs is that it can encapsulate both hydrophilic and hydrophobic molecules with similar loading efficiency owing to extensively available pores (Slowing et al., 2008; Desai et al., 2018; Xu et al., 2018). Majority of chemotherapeutics and nutraceuticals with anticancer potential are hydrophobic in nature and as is the case of MSNs have been frequently used to demonstrate delivery of such substances. RES is hydrophobic in nature and has been successfully loaded onto MSNs by our group previously (Ton et al., 2012; Summerlin et al., 2016). We demonstrated anticancer potential of RES in MCM-48 MSNs in colon cancer cell lines by improving their anti-inflammatory activity *in vitro* by reducing NF-κB (Summerlin et al., 2016). In addition, the effects of varying particle sizes i.e., 90, 150, and 300 nm with pore sizes of 3.5 and 7 nm on modulating aqueous solubility and anti-inflammatory activity of RES was studied (Juère et al., 2017).

Surface functionality is another property of MSNs which has significant effect on the biological performance of the encapsulated drug (Jambhrunkar et al., 2014). However, to date there has been no investigation of how RES interacts with MSNs with different surface chemistries and the effect of surface functionalities on release and biological activity of RES such as its anti-cancer potential. Herein, we hypothesize that MSNs functionalized with positively charged surface amine groups and negatively charged phosphonate groups loaded with RES may lead to effectively enhanced anti-cancer activity of RES in prostate cancer cells and reversed hypoxia induced Doc resistance in PC3 cells. In the present work, we have explored the effect of RES-loaded MSN to reverse the hypoxia-induced resistance to Doc in PC3 cell lines. The findings may help to establish use of preferential functional group and propose a platform nanoparticle-based drug delivery system for synergistic anti-cancer activity of RES with Doc for prostate cancer therapy.

## Materials and Methods

### Materials

Tetraethyl orthosilicate (TEOS, 99%), cetyltrimethyl ammonium chloride (CTAC, 25 wt% in H_2_O), triethanolamine (TEA, ≥ 99%), 3-aminopropyl-triethoxy silane (ATPES, 99%), 3-trihydroxysilyl-propyl methyl-phosphonate, mono-sodium salt (THMP, 50 wt% in H_2_O) were obtained from Sigma-Aldrich (Australia), Resveratrol (99% pure trans-Resveratrol) obtained from Candlewood Stars Inc. CT USA. All other reagents and solvents were of HPLC grade and did not require any further purification.

### Methods

#### Fabrication of Mesoporous Silica Nanoparticles

MSNs were synthesized following a previously established protocol with slight modifications (Pan et al., 2012). Briefly, 2 g CTAC and 0.04 g TEA were dissolved in 20 mL DI water and kept for vigorous stirring at 95°C in a silicone oil bath. After 1 h, 1.5 mL of TEOS was added dropwise at a rate of 1 mL per minute into the dispersion. This mixture was stirred for another hour under similar conditions. The mixture was allowed to cool, and the MSNs were collected as pellets after centrifugation (20,000 × g, 15 min). The particles were washed with ethanol several times and dried overnight at 60°C in an oven. The dried pellets were crushed and subjected to calcination in muffle furnace (Thermo scientific, Australia) at 550°C for 7 h to completely remove the surfactant.

#### Surface Modification of MSNs Particles

The MSNs were surface modified with two types of functional groups namely; (a) phosphonate (PO_3_) i.e., negative charge moiety and (b) amine (NH_2_) i.e., positive charged moiety. For phosphonate modification, 0.20 mL THMP was dissolved in 20 mL milli-Q water. The pH of solution was adjusted from 11 to ~5–6. The acidic pH prevents hydroxylation and condensation of silanol groups during the functionalization. To this pH adjusted solution, 200 mg of MSNs were added, and bath sonicated for 5 min. The mixture was refluxed overnight at 100°C and 1,000 RPM. Next day, the phosphonated MSNs (PO_3_-MSN) were collected as pellets by centrifugation (Multifuge X1R, Thermofisher, Australia) and washed two times with water followed by washing with ethanol (20,000 × g, 10 min each cycle). The pellets were oven dried overnight (60°C). For amine surface modification 250 mg MSNs were vacuum dried overnight to remove any moisture from the pores. The particles were then added into 50 mL toluene and stirred for 30 min (50°C). The temperature of silicone oil bath was increased to 115°C. APTES (262 μL) was added and the mixture was refluxed overnight. Next day, the amine modified MSNs (NH_2_-MSN) were collected by centrifugation and washed two times with acetone and once with ethanol. The pellets were oven dried overnight (60°C).

#### Resveratrol Loading Onto Surface Modified MSNs

Resveratrol was loaded onto surface modified MSNs using rotary evaporation method. For 10 wt% loading, 10 mg of RES were accurately weighed and dissolved in 8 mL methanol. The mixture was bath sonicated for 5 min. To this, 90 mg of MSNs were added and stirred overnight at room temperature. Next day, the organic solvent was evaporated using rotary evaporator at 40°C. The RES loaded particles were scratched off from round bottom flask (RBF) and stored in a dry cool place away from light.

#### Physicochemical Characterizations

The prepared particles before and after surface modifications were characterized for their particles size, surface charge, surface area, and pore size. The dynamic light scattering was performed using Nano series ZS instrument (Malvern, UK). The particles were suspended in PBS 7.4 and bath sonicated for 10 min prior to measurements. A Mettler-Toledo Thermogravimetric analysis (TGA)/differential scanning calorimetry (DSC) instrument was used to determine the functional groups incorporation in terms of percentage mass grafting. The particles were accurately weighed around 5 mg in the TGA alumina crucible (70 μL) and the method comprised of a temperature range of 50°C to 900°C with a temperature ramp rate of 10 degrees per minute under air ambient. The percentage loading capacity was also determined using thermogravimetry analysis. The particles were suspended in ethanol and sonicated for 10 min for TEM grid preparation and the images were obtained using a HITACHI HT7700B Transmission electron microscope operated at 100 kV. N_2_-Sorption analysis was done using a Tri-Star II 3020 Nitrogen adsorption system to measure the surface area and pore size of particles before and after RES loading onto functionalized MSNs. The particles were weighed around 60–70 mg and degassed prior to surface area analysis. Fourier Transformed Infrared (FTIR) Spectroscopy was performed using the Perkin Elmer FTIR spectrometer to confirm the incorporation of functional groups and Resveratrol onto the MSNs. The RES loading efficiencies (LE%) of the functionalized MSNs were calculated using equation described in **Section S1** (Supplementary Material).

#### *In vitro* Release Study

A calibration curve was prepared for RES ranging from a concentration of 100 ng mL^−1^ to 25 μg mL^−1^ (*R*^2^ = 0.999). Separate calibration curves were prepared for two different pH values i.e., pH 7.4 and pH 5.5 (Figure S5). The *in vitro* release experiments were performed by suspending 300 μg RES or equivalent of RES loaded particles in 5 mL of release medium (PBS 7.4 or PBS 5.5). At predetermined time intervals 1 mL was taken out, and centrifuged (20,000 × g, 5 min). The separated supernatant was diluted as per respective calibration curve and analyzed in HPLC at 306 nm. The volumes taken out at every time point were replaced with fresh buffer. The buffers used in this study were phosphonate buffers.

#### Proliferation Assay

PC3 cells were originally sourced from the American Type Culture Collection (ATCC). PC3 cells were cultured in RPMI1640 (1X) (Life Technologies) supplemented with 5% fetal bovine serum (FBS, Sigma). Culture media was replaced at 3/4-day intervals and at 70–80% confluency. Cells were passaged by washing twice with phosphate buffered saline (PBS) and detached with Trypsin/EDTA Solution (TE, Life Technologies). All cells were regularly tested for Mycoplasma by Mycoplasma detection KIT (Universal Mycoplasma Detection KIT, ATCC). Stock solution of the RES (10 mM) was prepared in dimethyl sulfoxide (DMSO) and stored at −20°C.

##### IncuCyte assay

Proliferation assays were performed using the IncuCyte live cell imaging system (Essen Biosciences, Australia). This system enables real-time cell counting by analyzing the occupied area (% confluence) of cell images over time. Briefly, 5 × 10^3^ cells were trypsinized and seeded in a Corning® 96 Well TC-Treated Microplates in 150 μL media and grown for 24 h. Next day, cells were treated transiently with RES and RES loaded MSNs (10, 15, and 20 μM) and placed in IncuCyte live cell imaging system as described previously (Matin et al., 2019). Two images per well were taken every 2 h for five consecutive days and confluency of the cells was measured. Each experiment consisted of three independent replicates. The cell viability was plotted and analyzed by the One-Way ANOVA test using GraphPad Prism software.

##### CyQuant assay

Cell viability assay was performed by measuring fluorescent quantification of DNA content by CyQuant NF assays (ThermoFisher Scientific, Catalog number—C35006). Briefly, 5 × 10^3^ cells/well-seeded in a black plastic plates (Perkin Elmer Life Sciences) in 150 μL media and grown for 24 h. After 24 h, cells were treated with RES, RES loaded and blank MSNs as controls (PO_3_-MSN and NH_2_-MSN) and placed in incubator at 37°C (5% CO_2_) for 72-h. After a 72-h period, cell viability was measured using CyQuant NF assays (ThermoFisher Scientific, Catalog number—C35006) according to the manufacturer's instructions and fluorescence (520 nm) was measured after excitation at 480 nm using a microplate reader (FLUO Star Omega, BMG LAB TECH). Each experiment consisted of three independent replicates. The cell viability was plotted and analyzed by the One-Way ANOVA test using GraphPad Prism software.

#### Presto-Blue Assay Under Hypoxic Conditions

Briefly, 5 × 10 ^3^ cells per well were seeded in a Corning® 96 Well TC-Treated Microplates in 150 μL media and grown for 24 h. After 24 h cells were treated with increasing concentrations of RES loaded MSNs, PO_3_-MSN-RES and Doc (Sigma Aldrich, Catalog number 01885) placed under normoxic (21% O_2_) and hypoxic conditions (2% O_2_) for 72-h. After a 72 h period, cell viability was measured using prestoBlue® Cell Viability Reagent (ThermoFisher Scientific, Catalog number–A13261) using a microplate reader (FLUO Star Omega, BMG LAB TECH) at excitation 560/10 nm and emission 590/10 nm. Each treatment was normalized to negative control analyzed by the One-Way test using GraphPad Prism software.

## Results and Discussion

### Physicochemical Characterizations

**Figure 1:**
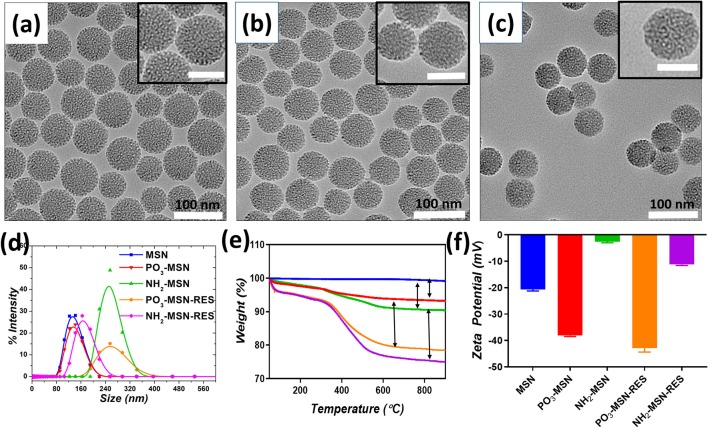
Transmission electron microscopy images of **(a)** Pristine MSN, **(b)** PO_3_-MSN, **(c)** NH_2_-MSN. The particles were suspended in ethanol and sonicated for 10 min for TEM grid preparation. The scale bar for inset images is 50 nm. Hydrodynamic diameter **(d)**. Intensity mean values for blank and RES loaded MSNs **(e)**. Thermogravimetric Analysis (TGA) plots for pristine and functionalized particles **(f)**. Zeta potential measurements plotted before and after RES loading onto MSNs. For DLS and zeta potential measurement 0.1 mg mL^−1^ dispersions of each particle were bath sonicated in PBS pH = 7.4 for 10 min prior to analysis.

MSNs with controlled and uniform size of ~60 nm were prepared using CTAC as structure directing agent, TEOS as silica precursor and TEA having dual action as basic catalyst and complexing/capping agent. TEA has been reported in earlier studies to control the particle size by limiting further growth of the particles due to its capping action as well as accelerating nuclei formation and ultimately yielding higher number of particles with similar sizes (Kobler et al., 2008; Lv et al., 2016). In our study, the TEA amount in the mixture was fixed at 0.04 g, which provided uniform spherical shaped MSNs with a controlled particle size of ~60 nm and porous structure as seen in TEM image, which is in agreement from previous findings (Figure 1a) (Pan et al., 2012). The surface of the MSN was functionalized with negatively charged phosphonate-functional groups (PO_3_-MSN) and positively charged amine-groups (NH_2_-MSN) using silane chemistry. Analysis of the TEM images shows that surface modification did not have any impact on size, shape, and morphology of the MSNs (Figures 1b,c).

The pristine and functionalized MSNs were further characterized by dynamic light scattering. Surface charge was monitored by determining zeta potential of the unloaded and RES loaded MSNs in PBS (pH 7.4). Pristine MSNs show a hydrodynamic diameter of 61 nm, which increased slightly to an average diameter of 72 nm after phosphonate modification. The NH_2_-MSNs however showed tendency to aggregate in PBS and had a higher value of hydrodynamic diameter i.e., 267 nm (Guillet-Nicolas et al., 2013) which was consistant with previous literature. The unloaded pristine MSNs show zeta potential of −20.7 mV, which was further increased to −38.1 mV after grafting of negatively charged functional groups i.e., the PO_3_ groups onto the MSN surface. The surface mass grafting of NH_2_ was confirmed by shift in zeta potential from −20.7 to −2.59 mV, which directly relates to amine functionalization (Table 1, Figure 1). The RES loading increased the magnitude of negative surface charge of the MSNs to −42.8 and −11.2 mV, respectively (Table 1, Figure 1).

**Table 1:** Particle size analysis of RES loaded and unloaded functionalized MSNs.

**Samples**	**PDI**	***Z*-average**	**Number mean (nm)**	**Intensity mean (nm)**	**Zeta potential**
MSN	0.325 ± 0.03	287.5 ± 40.23	61.85 ± 5.89	105.7	−20.7 ± 0.5
PO_3_-MSN	0.383 ± 0.06	376.0 ± 16.7	72.62 ±13.77	122.4	−38.1 ± 0.3
PO_3_-MSN-RES	0.368 ± 0.10	220.4 ± 3.75	59.65 ± 5.23	245.3	−42.8 ± 1.5
NH_2_-MSN	0.447 ± 0.14	1263 ± 852	267.3 ±14.03	255.4	−2.59 ± 0.3
NH_2_-MSN-RES	0.344 ± 0.02	345.6 ± 10.2	156.4 ± 2.83	194.6	−11.2 ± 0.3

**Figure 2:**
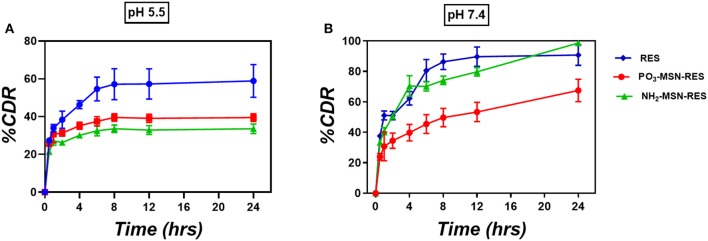
*In-vitro* release study. Three-hundred microgram RES and equivalent particles were weighed (10%wt. loading) and added into the release medium **(A)** PBS of pH 5.5 and **(B)** PBS of pH 7.4. At predetermined time points 1 mL was taken out from the release medium and centrifuged prior to analysis by HPLC. The volume taken out was replaced with fresh medium (*n* = 3 ± SD). The release is represented as percent cumulative drug released (%CDR) at any given time point.

The mass grafting of the functional groups and loading of RES onto MSNs was determined by TGA. Loading capacity, defined as the percentage loading of RES onto MSNs by weight, was also calculated using TGA, the values are presented in Table S1. The thermograms confirmed the functionalization of MSNs and loading of the functionalized MSNs with RES. As per Figure 1e, PO_3_ functional groups composed 4% of the total mass of the functionalized MSNs, while a higher grafting in case of NH_2_-MSNs with a value of 6.3% was found. The loading of RES was found close to theoretical loading i.e., 10% with 10.2, and 11.2% loading onto PO_3_-MSNs and NH_2_-MSNs, respectively (Table S1, Figure 2). Moreover, no endothermic melting peak of RES was observed for functionalized MSNs based RES formulation whereas, a clear endothermic melting peak (250 to 290°C range) is visible for free RES. This indicates that the drug is potentially inside the pores as it is maintained in its amorphous form, while free RES exists in crystalline form (Figure S1). Surface functionalization of the MSNs was also confirmed using FTIR spectroscopy, which displayed IR vibrational bands specific to each functionalization (Figures S2a,b). The pristine MSNs present peaks at 1,080 cm^−1^ and 810 cm^−1^ corresponding to Si-O and Si-O-Si vibrations of silanol groups. Salinization with NH_2_ and PO_3_ can be confirmed by the appearance of peaks between 2,800 cm^−1^ and 2,980 cm^−1^ corresponding to stretching vibrations of C-H bond of the propyl chain of the silane (Kumeria et al., 2013). Fourier Transformed Infrared (FTIR) spectrum of pure RES showed a typical trans-olefinic band at 961 cm^−1^ and band of O-H stretching at 3,202 cm^−1^. The three characteristic bands of RES can be seen in RES loaded MSNs ensuring the successful mass grafting of RES onto the functionalized MSNs. These three characteristic bands are shown in Figures S3a,b where, 1,380, 1,580, and 1,606 cm^−1^ bands correspond to C-O stretching, C-C olefinic stretching and C-C aromatic double bond stretching (Apoorva et al., 2014).

The porosity and surface area of pristine and functionalized MSNs were assessed by nitrogen physisorption analysis. Figure S4 shows MSN had a pore size of 2.7 nm, which remained largely unaffected by functionalization and drug loading confirming that particles still retain majority of their porous architecture after functionalization and RES loading. It is important to note that even after 10% loading we didn't see any significant change in pore size however both surface area and pore volume decreased. This could be due to the hydrophobic nature of RES which will repel from hydrophilic functional groups on the surface of the pores and potentially exist as isolated amorphous aggregates (Abbaraju et al., 2014). The BET surface area of pristine MSNs was found to be 397 m^2^/g, which decreased after PO_3_- and NH_2_- surface modifications (Table 2). The available surface area was further reduced to 295, 119, and 74 m^2^/g after loading of RES, indicative of occupation of higher number of pores with RES loaded onto MSNs.

**Table 2:** Surface area and porosity of MSNs after functionalization and RES loading.

**Samples**	**BET surface area (m^**2**^/g)**	**Pore size (A^**°**^)**
MSN	397.60	22.99
PO_3_-MSN	221.24	22.68
NH_2_-MSN	165.67	18.33
PO_3_-MSN-RES	119.15	21.66
NH_2_-MSN-RES	73.92	22.65

### *In vitro* Release Profile of RES From Functionalized MSNs

The *in vitro* release profile of RES from functionalized particles is shown in Figure 2 as cumulative % release. At pH 7.4 free RES was released up to 85% in first 8 h followed by 100% release in 24 h. No significant control over the release was observed for NH_2_-MSNs at pH 7.4 as the significant content from the particle was released within first 8 h similar to free drug. PO_3_-MSNs, however, showed control over release with only 40% RES was released in first 8 h followed by up to 65% release over 24 h. At pH 5.5 (which loosely mimic the pH inside the cancer cell) only 60% of the free RES is released over 24 h whereas PO_3_-MSNs and NH_2_-MSNs show 40% of total release in 24 h. The overall release behavior could be attributed to kinetic solubility, which is affected by dissolution media and needs to be investigated in detail.

### *In vitro* Assessment of Anti-cancer Potential of Resveratrol in PC3 Cell Line

**Figure 3:**
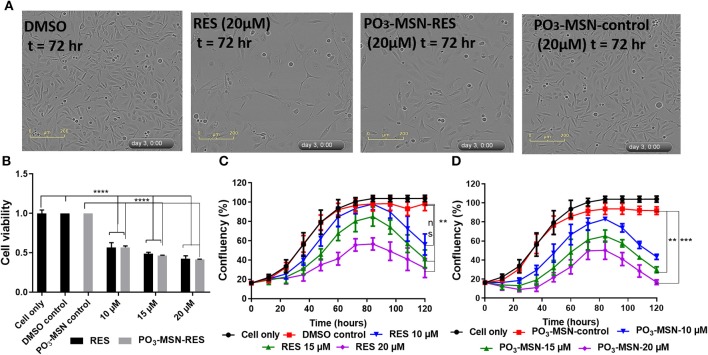
Resveratrol and PO_3_-MSN-RES inhibited proliferation and cell viability of PC3 cells. Cell proliferation and cell viability were measured using the InCucyte live system and CyQUANT, respectively. Data is presented as Mean ± SEM, *n* = 3, One-way ANOVA test, Tukey's *post-hoc* test, **P < 0.01, ***P < 0.001, **** *P* < 0.0001. **(A)** Representative phase contrast images of DMSO control, 20 μM of RES, 20 μM of PO_3_-MSN-RES and 20 μM of PO_3_-MSN-control treated PC3 cells are shown at 72 h. **(B)** RES and PO_3_-MSN-RES inhibited cell viability in a dose dependent manner (Each treatment normalized to vehicle i.e., DMSO and PO_3_-MSN-Control). **(C)** RES inhibited proliferation in PC3 cells **(D)** PO_3_-MSN-RES reduced proliferation.

RES reduced proliferation of PC3 cells in a dose dependent manner and 20 μM showed a significant retardation in proliferation of the cells (Figure 3A) compared to DMSO treated cells as shown in Figure 3C. The IC_50_ value was calculated to be 14.86 μM for free RES. Further, 10, 15, and 20 μM of RES significantly reduced cell viability of PC3 cells in a dose dependent manner compared to DMSO control as measured using the Presto-Blue assay (Figure 3B).

Afterwards, PO_3_-MSNs and NH_2_-MSNs were tested to see the variation of anti-proliferative activity of RES encapsulated within these particles. A preliminary analysis showed that NH_2_-MSNs had an IC_50_ of 20.45 μM at 72 h i.e., higher than that of free RES while PO_3_-MSN-RES indicated better anti-proliferative activity than that of free RES (Figure S6). A detailed characterization by using PO_3_-MSN-RES showed an IC_50_ of 7.15 μM indicating enhanced anti-proliferative potential. The proliferation of PC3 cells was reduced in a dose dependent manner and 15 and 20 μM of PO_3_-MSN-RES significantly reduced proliferation of PC3 cells (Figures 3A,D) compared to DMSO dissolved RES. Further, 10, 15, and 20 μM of PO_3_-MSN-RES significantly reduced the cell viability of PC3 cells (Figure 3B), similar to RES as measured using the CyQuant assay. It is to be noted that the blank MSNs had no effect on cell proliferation (Figures 3A,D, Figure S6). The findings confirmed that using the PO_3_ modified colloidal MSNs improves the biological activity of RES as evident by significant decrease in IC_50_ of RES from 14.86 μM DMSO dissolved RES to 7.15 μM for the PO_3_ modified MSNs, a 2-folds reduction compared to RES.

### *In vitro* Assessment of Synergistic Effects of Resveratrol and Docetaxel

RES is known for its anti-cancer potential through synergistic effects with chemotherapeutic agents such as doxorubicin (Kim et al., 2014). An interesting feature of RES reported recently, is that it enhances the chemo-sensitivity of doxorubicin (DOX) in breast cancer cells. It increases the cellular influx of DOX due to down-regulation of ATP binding transporter genes MDR-1 and MRP-1 (Kim et al., 2014). In the present study, an attempt was made to understand if there is possibility to reduce the Doc-resistance using RES encapsulated within MSNs. Because of resistance, higher amounts of Doc are required for desired therapeutic action and Doc is typically cytotoxic to normal cells. The tumor micro-environment plays a substantial role to cause resistance against taxanes. Particularly, hypoxia renders the prostate cancer cells resistant to drugs acting on cell cycle by retarding cell proliferation and also leads to promotion of malignant cell phenotype (Lohiya et al., 2016). In our study, a combination of the lowest RES concentration i.e., 10 μM with a range of concentration of Doc was tested to see if there is a synergistic effect of RES in hypoxia induced chemoresistance of prostate cancer cell lines. The experiments were conducted using normoxia and hypoxia conditions to observe cell viability.

**Figure 4:**
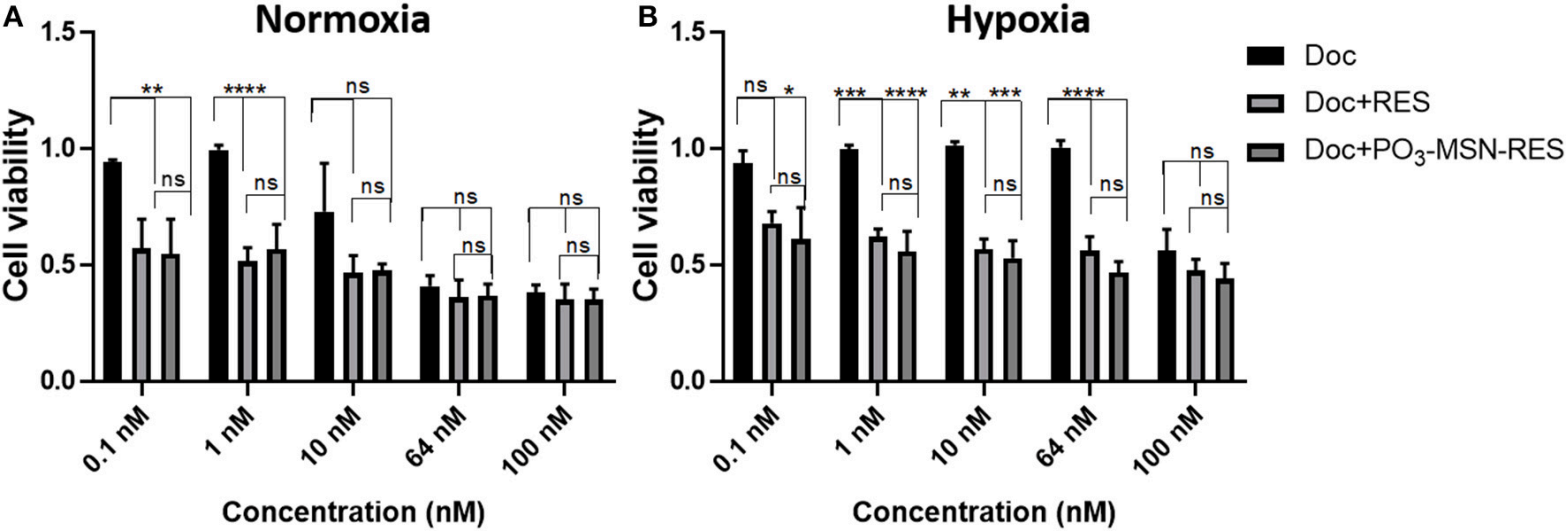
Inhibitory effect of RES and PO_3_-MSN-RES on hypoxia-induced resistance to Doc in PC3 cells. PC3 cells were cultured with increasing concentrations of Doc (0.1–100 nM), RES, PO_3_-MSN-RES in **(A)** normoxia and **(B)** hypoxia for 72 h and cell viability was determined. Hypoxia induced docetaxel resistance in PC3 cells and docetaxel sensitivity was enhanced after combination with RES and PO_3_-MSN-RES under hypoxic conditions in PC3 cells. Cell viability was normalized to DMSO and PO_3_-MSN-Control. Data was presented as Mean ± SEM, *n* = 3, One-way ANOVA test, Tukey's *post-hoc* test, ****P < 0.0001, ***P < 0.001, **P < 0.01, *P = 0.0177.

A higher cell viability was observed in the hypoxic conditions for the same dose of treatment as compared to normoxic conditions for a dose range of 0.1–100 nM of Doc, validating the effects of hypoxia induced Doc resistance. However, the hypoxic cell viability was significantly reduced by Doc + RES and Doc + PO_3_-MSN-RES as compared to free Doc used alone (Figure 4). The effect was pronounced up to a concentration of 100 nM and indicated that RES could reduce the concentration of Doc required to achieve the significant reduction in cell viability. It could be seen that the lower concentrations of Doc (0.1–10 nM) in combination with RES (10 μM) were enough to reduce the cancer cell burden to half (Figure 4). The effects of RES to reduce Doc concentration was in agreement from previous study with nano-encapsulated RES to reduce resistance against Doc (Singh et al., 2018). The synergistic effect can be due to substantial cytotoxicity due to its anti-oxidant potential which helps in scavenging the reactive oxygen species (ROS) generated due to hypoxia (Shimojo et al., 2013). However, the magnitude of effects of PO_3_-MSN-RES paralleled and sometimes exceeded the effects induced by equimolar concentrations of free RES dissolved in DMSO. These findings indicate no loss of biological activity when RES in encapsulated within PO_3_-MSNs. It is important to mention that the empty nanoparticles alone induced no effect on cell viability or proliferation indicating that particles alone are relatively inert.

## Conclusion

In this study, we have successfully loaded functionalized MSNs with RES and investigated the effect of surface functionality on physicochemical characteristics, drug loading, *in vitro* release and *in vitro* anti-proliferation and cytotoxic potential using prostate cancer cell line. Both positive and negatively charged MSNs showed efficient encapsulation (~100% loading) and no sign of crystalline resveratrol after encapsulation which was confirmed using DSC. *In-vitro* release assay revealed that compared to free RES and NH_2_-MSNs negatively charged PO_3_-MSNs showed superior control over release at both pH 7.4 and pH 5.5. Consequently, PO_3_-MSNs demonstrated significantly higher anti-proliferative activity compared to free RES (pre dissolved in DMSO) and NH_2_-MSNs in PC3 cells. The free RES (pre-dissolved in DMSO) and RES encapsulated in PO_3_-MSNs reversed the hypoxia induced resistance to Doc in PC3 cells, indicated by lower concentrations of Doc required to achieve >50% cytotoxicity as compared to Doc alone. In summary, our results indicate that functionalized silica based nanoparticles can be used to combine polyphenols (such as RES) and chemotherapeutic agents which are prone to drug resistance (Doc) in order to improve the efficacy of both polyphenol and chemotherapeutic drug in resistant cancers such as prostate cancer. However, further testing in other prostate cancer cell lines and *in vivo* efficacy tests are warranted in order to translate these formulations before they can be tested clinically.

## Data Availability

The raw data supporting the conclusions of this manuscript will be made available by the authors, without undue reservation, to any qualified researcher.

## Author Contributions

ZC, SS, GK, TK, JB, and AP contributed conception, design, and significant writing. ZC and SS performed majority of experiments. ZQ performed TEM analysis. TJ and MA helped in statistical analysis, and critically analyzed the manuscript figures and provided suggestions. All authors contributed to manuscript revision, read, and approved the submitted version.

### Conflict of Interest Statement

The authors declare that the research was conducted in the absence of any commercial or financial relationships that could be construed as a potential conflict of interest.
