# Supplementary material for: Encapsulation and Controlled Release of Resveratrol Within Functionalized Mesoporous Silica Nanoparticles for Prostate Cancer Therapy
Source: Front Bioeng Biotechnol. 2019 Sep 18;7:225. doi: 10.3389/fbioe.2019.00225 (PMC6759778; doi:10.3389/fbioe.2019.00225)
Supplement: Supplementary file 1 [file Table_1.DOCX]

Supplementary Material

**Encapsulation and Controlled Release of Resveratrol within Functionalised Mesoporous Silica Nanoparticles for Prostate Cancer Therapy**

Zanib Chaudhary,^a,d^**^†^** Sugarniya Subramaniam,^b,c^**^†^** Gul Majid Khan,^d^ Muhammad Mustafa Abeer,^a^ Zhi-Qu,^a^ Taskeen Janjua,^a^ Tushar Kumeria,^a,c,e^* Jyotsna Batra,^b,c^* Amirali Popat ^a,c,e^*

^a^School of Pharmacy, The University of Queensland, Brisbane QLD 4072, Australia

^b^School of Biomedical Sciences; Faculty of Health, Institute of Health and Biomedical Innovation, Australian Prostate Cancer Research Centre-Queensland (APCRC-Q), Queensland University of Technology, Brisbane, Australia

^c^Translational Research Institute, Woolloongabba, Queensland-4102, Australia

^d^Department of Pharmacy, Quaid-i-Azam University, Islamabad-45320, Pakistan

^e^Mater Research Institute, Woolloongabba, Queensland-4102, Australia

**^†^**The authors contributed equally

*** Correspondence:**Corresponding Author
[a.popat@uq.edu.au](mailto:a.popat@uq.edu.au), [jyotsna.batra@qut.edu.au](mailto:jyotsna.batra@qut.edu.au) and t.kumeria@uq.edu.au

Postal Address: Pharmacy Australia Centre of Excellence, 20 Cornwall Street, Woolloongabba, Queensland-4102, Australia

***Supporting Materials***

**SECTION S1. Calculation of RES Loading efficiency**

The loading efficiency was calculated using the following equation.

$\left( \boldsymbol{b} \right) \boldsymbol{\% Loading Efficiency}=\frac{Mass(drug loaded onto the particles)}{Mass(Initial drug added from stock)}\times100$

e.g. for PO_3_-MSN-RES $\% Loading Efficiency =\frac{10.2mg}{10.2mg}\times100=100 \%$

**Table S1: Calculation of % mass grafting/ %LC from TGA plots**

| Samples | Mass Grafting | % LC | %LE |
| --- | --- | --- | --- |
| MSN | 0.4 | -- |  |
| PO_3_-MSN | 4 | 10.2 | 102 |
| NH_2_-MSN | 6.3 | 11.2 | 103 |

**Figure S1**: **Diffraction Scanning Calorimetry** (DSC) plots for free RES and Functionalised MSNs based RES formulations.


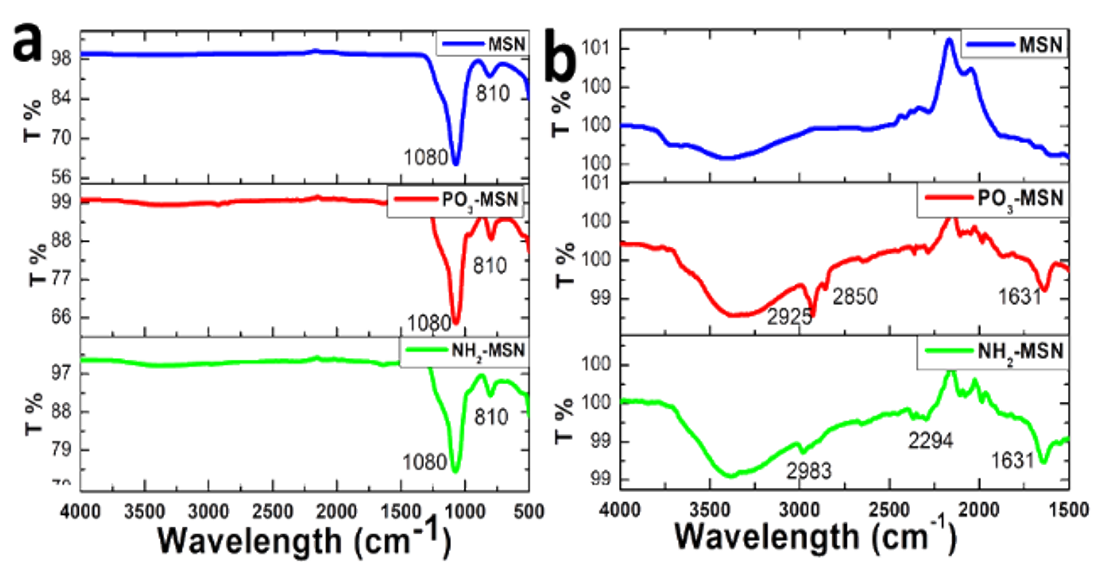


**Figure S2. Fourier Transform infrared (FTIR) spectroscopy** of Blank MSNs (without RES) (a). MSN, PO_3_-MSN and NH_2_-MSN for range 500 cm^-1^ to 4000 cm^-1^. (b). IR spectra for the respective particles for a range 1500 cm^-1^ to 4000 cm^-1^.


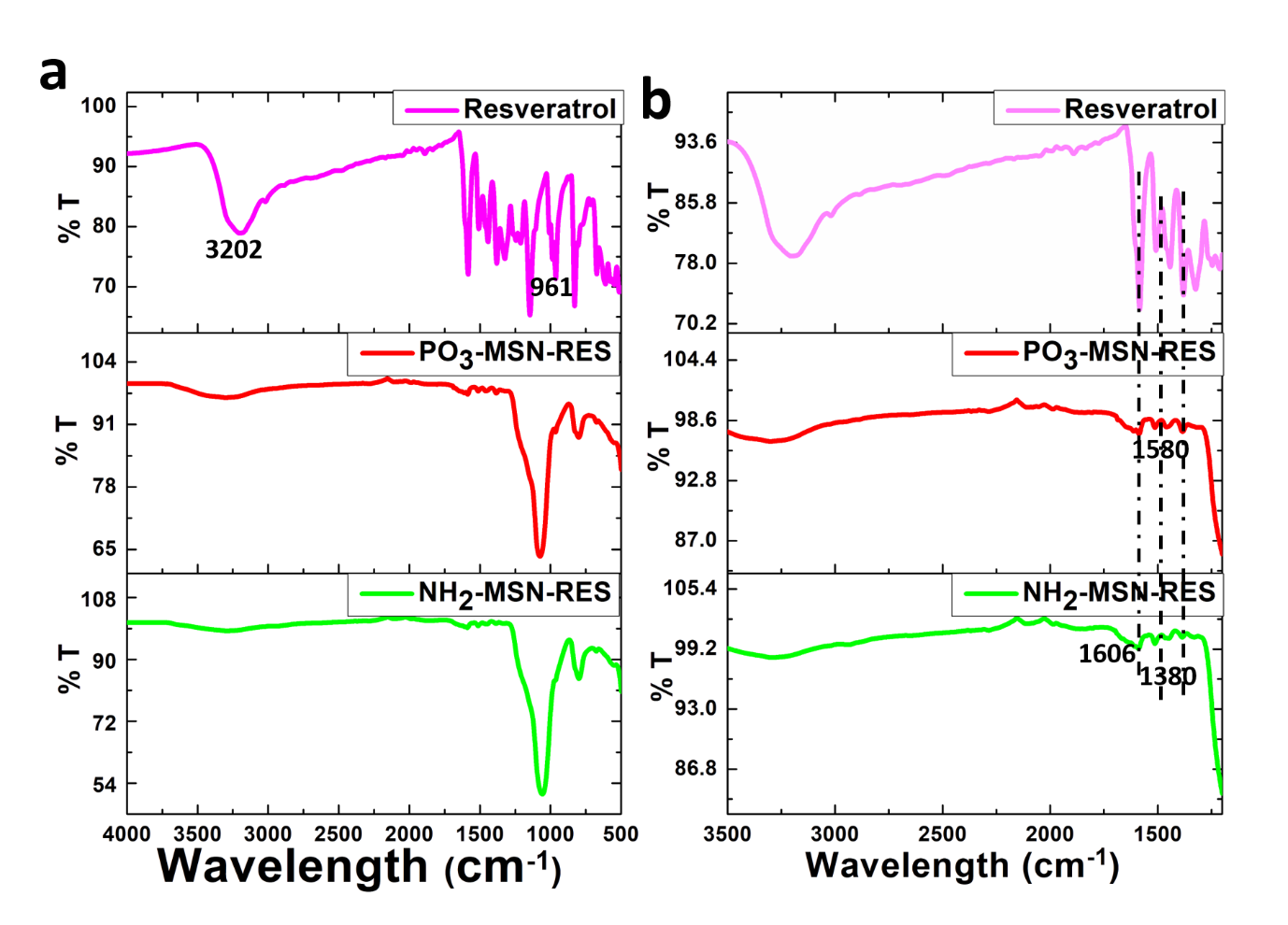


**Figure S3. Fourier Transform infrared (FTIR) spectroscopy** of RES and loaded MSNs (a). RES, PO_3_-MSN-RES and NH_2_-MSN-RES for range 500 cm^-1^ to 4000 cm^-1^. (b). IR spectra for the respective particles for a range 1200 cm^-1^ to 3500 cm^-1^.


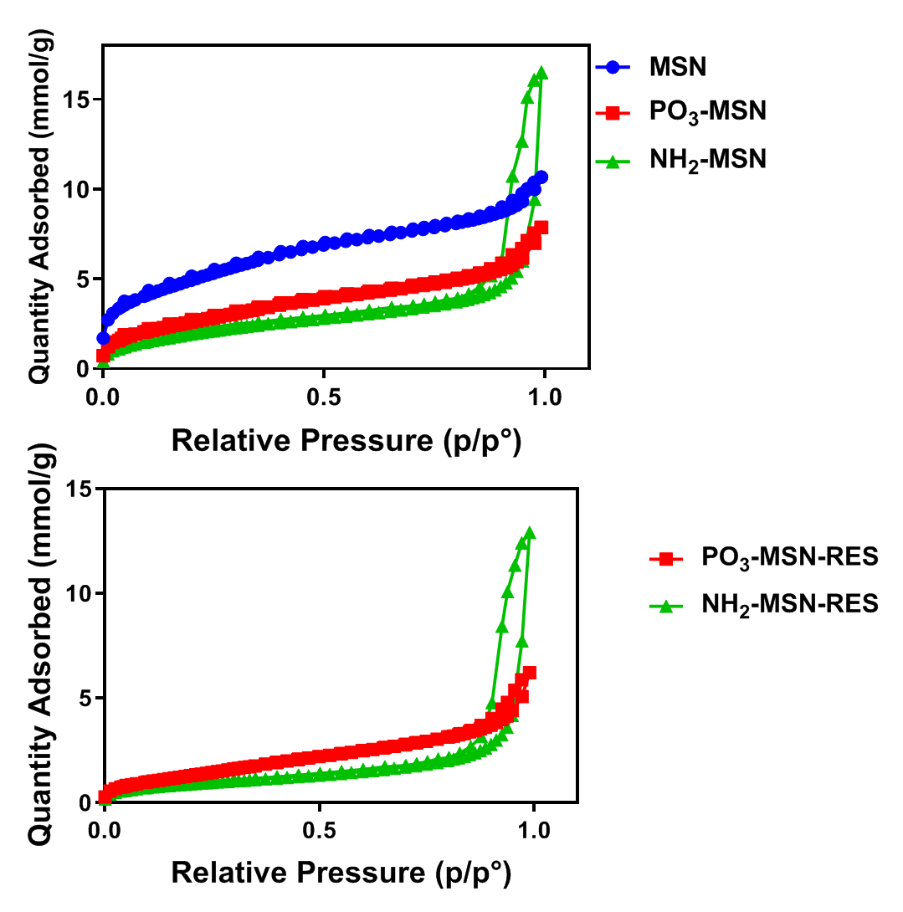


**Figure. S4 Nitrogen physisorption Analysis.** The samples were degassed for 24 hours prior to analysis to remove any air bubble or moisture from the pores. The particles show hysteresis type 4. The particles retain their porous architecture after functionalisation and RES loading. The surface area, however, is decreased after surface functionalisation and drug loading as observed in previous studies as well.

**Resveratrol HPLC Standard curves for quantification of RES at pH 7.4 and pH 5.5**

**Figure S5.** The HPLC Calibration curves for RES. The calibration curves were drawn using a concentration range of 100ng mL^-1^ to 25 µg mL^-1^. The stock of RES was prepared in HPLC grade methanol (100 %). The dilutions were then prepared to make the final concentration in a solvent mixture of Methanol and PBS in a ratio of 90:10.


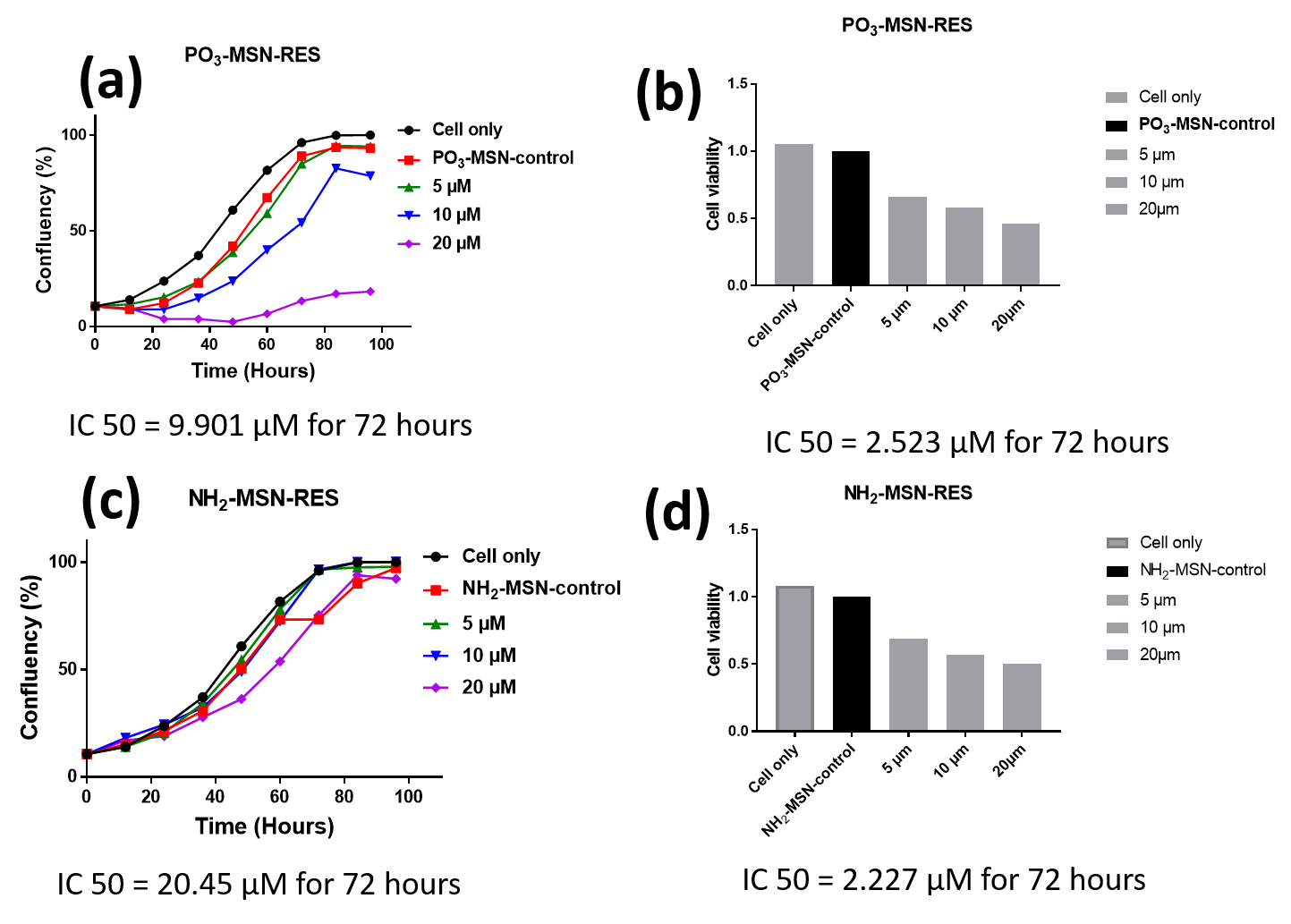


**Figure S6: *PO_3_-MSN-RES and NH_2_-MSN-RES inhibited cell viability (b, d) and proliferation (a, c) of PC3 cells*.** a) PC3 cells were treated with increasing concentrations of PO_3_-MSN-RES and NH_2_-MSN-RES. Cell proliferation was measured using the InCucyte live system and each treatment normalized to vehicle (Blank). PO_3_-MSN-RES and NH_2_-MSN-RES inhibited PC3 cell viability and proliferation in a dose dependent manner Note: All the experiments were performed in triplicates (n = 3).
